# Supplementary material for: Impact of Palliative Care Services on Treatment and Resource Utilization for Hepatorenal Syndrome in the United States
Source: Medicines (Basel). 2021 May 12;8(5):21. doi: 10.3390/medicines8050021 (PMC8150700; doi:10.3390/medicines8050021)
Supplement: Supplementary file 1 [file medicines-08-00021-s001.zip › medicines-1202332-supplementary.pdf]

Supplementary Materials: Impact of Palliative Care Services on Treatment and Resource Utilization for Hepatorenal Syndrome in the United States

Charat Thongprayoon, Wisit Kaewput, Tananchai Petnak, Oisin A. O'Corragain, Boonphiphop Boonpheng, Tarun Bathini, Saraschandra Vallabhajosyula, Pattharawin Pattharanitima, Ploypin Lertjitbanjong, Fawad Qureshi and Wisit Cheungpasitporn

Table S1. ICD 9 CM codes.

Hepatorenal syndrome	572.4
Palliative care service	V66.7
Smoking	305.1, 649.0, 989.84
Alcohol drinking	291.0, 291.1, 291.2, 291.3, 291.4, 291.5, 291.8, 291.81, 291.82, 291.89, 303.00–303.03, 303.90–303.93, 305.00–305.03
Anemia	283.00-285.9
Obesity	278.0, 278.00, 278.01, 649.10–649.14, 793.91, V85.30–V85.4, V85.54
Diabetes Mellitus	249.00-249.31, 249.40–249.91, 250.00–250.33, 250.40–250.93, 648.00–648.04, 775.1
Hypertension	401.xx, 402.00–405.99, 437.2, 642.00–642.24, 642.70–642.94
Dyslipidemia	272.xx
Congestive heart failure	428.xx
Chronic kidney disease	585.1, 585.2, 585.3, 585.3, 585.4, 585.5, 585.6, 585.9
HBV cirrhosis	070.2, 070.20, 070.21, 070.22, 070.23, 070.3, 070.30, 070.31, 070.32, 070.33, 070.42, 070.52(hepatitis B)+571.5(cirrhosis)
HCV cirrhosis	070.41, 070.44, 070.51, 070.54, 070.7, 070.70, 070.71 (hepatitis C)+571.5 (cirrhosis)
Alcoholic cirrhosis	571.2
Biliary cirrhosis	571.6
Non-alcoholic steatohepatitis	571.8, 571.5, and no codes for hepatitis B or hepatitis C
Hepatocellular carcinoma	155.0
Palliative care	V66.7
Do not resuscitate status	V49.86
Atrial flutter/fibrillation	427.31, 427.32
Coronary artery disease	410.xx, 411.xx, 412.xx, 413.xx, 414.xx
Ventricular arrhythmia /Cardiac arrest	427.1, 427.41, 427.5
Invasive mechanical ventilation	96.70–96.73
Non-invasive ventilation	93.90
Renal replacement therapy	39.95, v45.1, v56.0, v56.1, 54.98, v56.2, v56.32
Blood transfusion	99.00–99.07
Enteral nutrition	96.6
Paracentesis	54.91
Gastrointestinal bleeding	456.0, 456.20, 530.82, 531.00–531.41, 531.60, 531.61, 532.00–532.41, 532.60–532.61, 533.00–533.41, 533.60, 533.61, 534.00–534.41,534.60, 569.3, 578.0, 578.1, 578.9

Bloodstream infections	003.1, 036.2, 036.42, 038.4, 038.40, 038.41, 038.42, 038.43, 038.44, 038.49, 038.3, 038.0, 038.1, 038.10, 038.11, 038.12, 038.19, 038.2, 112.5, 112.81, 117.9, 038.8, 038.9, 449, 790.7, 995.92
Septic shock	785.52
Acute organ dysfunction	
Acute kidney injury	584, 584.5, 584.6, 584.7, 584.8, 584.9 (exclude 585.5, 585.6)
Respiratory failure	518.81, 518.82, 518.85, 786.09, 799.1, 96.7, 96.70, 96.71, 96.72
Circulatory failure	458.8, 458.9, 785.5, 785.50, 785.51, 785.52, 785.59, 796.3
Liver failure	570, 572.2, 573.3, 573.4
Neurological failure	293, 293.0, 293.1, 293.8, 293.81, 293.82, 293.83, 293.84, 293.89, 293.9, 348.1, 348.3, 348.30, 348.31, 780.01, 780.09, 48.39, 89.14
Hematologic failure	286.6, 286.7, 286.9, 287.49, 287.5
